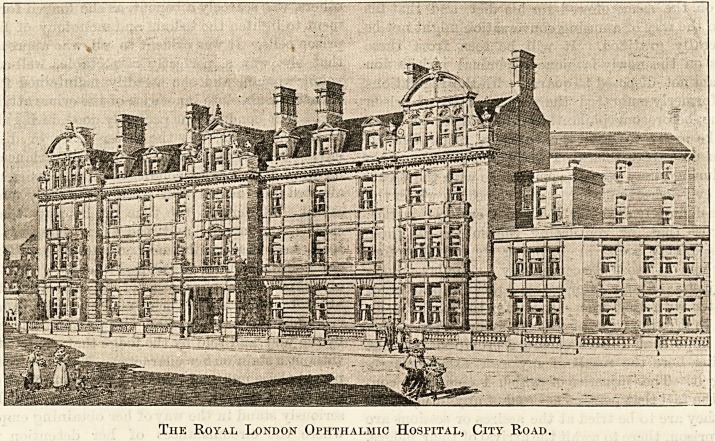# The Oldest Eye-Hospital in England

**Published:** 1899-07-29

**Authors:** 


### The Institutional Workshop.

### THE OLDEST EYE-HOSPITAL IN ENGLAND.

### A CHAT WITH THE SECRETARY IN THE NEW BUILDING.

BY OUR COMMISSIONER.

Next month the patients at the Royal London Ophthalmic Hospital will be transferred from Moorfields—95 years after the time of its foundation—to the handsome new home of the ancient institution in the City Road, which was formally opened by the Duke and Duchess of York on June 27th. A brief description of the buildings was given in The Hospital at the time of that function, but I have since had an opportunity of inspecting them under the guidance of Mr. Robert Bland, the secretary, and of discussing the position and prospects of the charity. Except the workmen, we had the place to ourselves, but things were being rapidly got in a state of order, and I was able to form a very fair opinion of what the hospital will be like when it is in full working order.

The Royal London Ophthalmic Hospital, City Road.

Beginning with the basement, Mr. Bland conducted me into the bedding, linen, meat, grocery, dispensary, and general store rooms, a box-room for the resident staff, and the stores receiving office. We also passed through the boiler-room, peeped into the motor-room, and then, ascending to the top floor, worked our way downwards. There are four floors, the fourth being the sick nurses' quarters. These are quite shut off, and are very roomy and adequate. On the third floor, the first and most important rooms are, of course, the wards (one is called the Princess May), which accommodate 30 patients. Here, as throughout, I was struck with the care and thought which have been manifested in all the details of the arrangements. The colouring is soft to the eye. Each patient is provided with a locker, in which he can place his toilet requisites. Lockers outside the wards are provided for clothing. All the furniture, too, is in the best taste, down to the new coal-boxes designed by the matron. Patients suffering from diseases of the eye are often able to be about during the day, and a pleasant, airy day-room has, therefore, been provided on each floor for them. In spite of the hospital being surrounded by buildings, the architects have made it wonderfully light. In the children's ward are pretty cots; friends have provided a rocking-horse and other toys for the little ones. The kitchen is on this floor, and, as Mr. Bland pointed out, all the cooking is done by gas or by steam, no coal being used. The milk for patients is sterilised, and there are dinner-wagons to convey the food downstairs. The nurses are partly provided for on the third floor, and the servants entirely. There is a large dining-room for the former, and excellent bed-rooms. Each nurse has a separate room, and all the furniture is fixed. The quarters of the night nurses are divided by a swing door from those of the day nurses, in order that the former may not be disturbed. Close to the night nurses' rooms is a fire escape and outside staircase, while, as a further provision in the event of fire, there are nine hydrants in the buildings.

The wards on the second floor accommodate 56 patients, and there is a smoking-room for the men. Here, also, is the nurses' cheerful sitting-room, with its comfortable window seats. The theatre and anæsthetic-room particularly claim attention. The floor of the theatre is made of terrazzo, the walls and ceiling are of opalite, the shelves of glass and metal. The blackboard is likewise made of glass, and there is a special blind made of painted cloth. The doors are solid, without ledges for dust to collect on, the great object being to keep everything as clean as possible so that the operations shall be made done the most favourable conditions.

Proceeding to the first floor we passed along wards with beds for 52 patients, and through the rooms for the matron, house surgeons, and rest of the staff, to a separate wing in which are the curator's room, laboratory, and museum. The students under the curator have access by means of a side staircase to the second floor. On the ground floor, which contains the very important department for out-patients, are the ophthalmoscope-room, the largest of the kind in the United Kingdom, the lecture-room, and the library, with the refraction-room, and the consulting-room. Here likewise are the dispensary, the X-ray room, and the spectacle-room. Some idea of the nature of the work going on may be gathered from the fact that, besides the surgeons and assistant surgeons, there are some 60 chief clinical and clinical assistants attached to the hospital.

"And how many patients?" I asked the secretary, as, having traversed the convenient administrative offices, we sat down in the board-room for a chat.

"About 400 every day," he replied. "You know," Mr. Bland continued, "that the chief feature of the work at Moorfields has been the out-patient department, in which each surgeon has had his desk. Here the out-patient department has been designed especially for the requirements of this large number of patients. The questions of light and cleanliness have been carefully studied. We think the refraction-room a marvel of light."

"How many patients can be examined at one time in the ophthalmoscope-room?"

"Eighteen. You noticed, perhaps, that care has been taken by the architects to prevent patients on their way from the waiting to the consulting rooms from crossing the path of others who are entering the building. Again, as to the spectacle-room: the optician appointed to the hospital attends there daily. I may mention that patients who are not able to pay the full price of spectacles are helped by the hospital. Sometimes the entire cost is defrayed, but this is not done until the inquiry officer has satisfied himself that the patient is unable to pay. The hospital is absolutely free to poor persons suffering from disease of the eye, and no letters of recommendation are required."

"You might tell me something about the X-ray room."

"As you know, X-ray work proves an important factor nowadays in ophthalmic surgery. If chips of metal get into the eye and the wound heals up, the position of the foreign body is located by means of the Röntgen rays, and it is extracted by a magnet for which we have a special electric current."

"You have admirable provision for the nurses?"

"Yes, it is in striking contrast to that at Moorfields. The object of Miss Robinson, the matron, who has had a great deal to do with the provision for nurses, has been to make things comfortable without being luxurious. In furnishing and fitting throughout she has taken much care to obtain not only what is useful, but also what will please the eye. The committee, who are most business-like in their management of the hospital, have, of course, had a voice in all the arrangements. The details of the furnishing have been supervised by the chairman of the Building Committee, who has devoted a great deal of time and attention to this matter."

"I see that you intend to have a chapel on the ground floor?"

"Eventually; but it has been impossible to do anything as yet. Even the walls are not plastered. Not a penny of the money given to the maintenance of the hospital is devoted to the chapel, the completion of which is dependent on donations for this express purpose. When finished it will be available for organ recitals. Our patients, not being allowed to read, have a monotonous time while in hospital, and much appreciate music."

"I conclude that the removal means enhanced expenditure; how are you going to face the prospect?"

"Our annual expenditure at Moorfields was about £8,000. As far as we can estimate it in the City-road, without actual experience of the new buildings, I do not think it will be less here than £12,000. The principal item in this difference is the ground rent of £1,210. At Moorfields the property was almost entirely freehold, and the sale of it, together with subscriptions, will almost defray the cost of these buildings. We are not appealing for assistance for the latter, but we do most urgently appeal to the public for increased annual subscriptions and donations to meet the increased expenditure, and for special donations in order to form a fund the interest of which will pay the heavy annual rent."

"Why will the expenses be 50 per cent more?"

"The building is much larger. The area of the four floors is upwards of three acres. There is a larger nursing staff, and a considerable increase in the staff of servants, owing to the large increase of in-patients. In these days of close scrutiny it is of vital importance that the staff should be efficient."

"What are your views about the alteration of site?"

"I must admit that at first City Road does not sound as accessible as Moorfields. On the other hand, a great number of patients come from the country, and these buildings are within easy reach of St. Pancras and King's Cross by tram; while they are only a very short distance from Liverpool Street. The new hospital is in close contact with a densely populated district, from which many of the patients come. My chief objection to the new site is on account of the deafening noise from the traffic. I hope, however, that this may be remedied very soon. The various hospitals in the City Road have approached the St. Luke's Vestry with the object of substituting a noiseless pavement, and they have promised to give the matter their best consideration."

## Figures and Tables

**Figure f1:**